# Impact of the Exposome in Type 1 Diabetes: Protocol for a Scoping Review

**DOI:** 10.2196/73424

**Published:** 2025-07-17

**Authors:** Maurane Rollet, Gloria A Aguayo, Jean-Pierre Riveline, Emmanuel Cosson, Guy Fagherazzi

**Affiliations:** 1 Deep Digital Phenotyping Research Unit Department of Precision Health Luxembourg Institute of Health Strassen Luxembourg; 2 Faculty of Science, Technology and Medicine University of Luxembourg Esch-sur-Alzette Luxembourg; 3 Immediab Lab Hôpital Necker-Enfants Malades Paris France; 4 Department of Diabetes Lariboisière Hospital Assistance Publique – Hôpitaux de Paris Paris France; 5 Nutritional Epidemiology Research Team (EREN) Université Sorbonne Paris Nord and Université Paris Cité Centre de Recherche Épidémiologie et Statistique Paris France; 6 Department of Endocrinology-Diabetology-Nutrition, AP-HP, Avicenne Hospital Université Paris 13, Sorbonne Paris Cité, CRNH-IdF, CINFO Bobigny France

**Keywords:** type 1 diabetes, environmental exposure, pollutants, autoimmune disease, diabetes onset, diabetes complications

## Abstract

**Background:**

The recent increase in the incidence of type 1 diabetes (T1D) over the past few decades has been too rapid to be explained solely by genetic factors. It is likely influenced by environmental factors as well. Understanding these environmental factors, the exposome, is essential for identifying new disease prevention and management strategies.

**Objective:**

This paper aims to present a protocol for a scoping review to gather current knowledge on the various dimensions of the exposome relevant to the development and outcomes of T1D, highlight existing knowledge gaps, and provide a foundation for future research.

**Methods:**

We will follow principles from the Joanna Briggs Institute (JBI) methodology for scoping reviews and the PRISMA-ScR (Preferred Reporting Items for Systematic reviews and Meta-Analyses extension for Scoping Reviews) guidelines. We will search for relevant publications in 5 databases: PubMed, Embase (Elsevier), CINAHL (EBSCO), Web of Science (Clarivate), and Google Scholar. All studies on the impact of environmental exposures on T1D development or acute (hypoglycemia or hyperglycemia) and chronic (cardiovascular disease, retinopathy, neuropathy, and others) T1D complications will be collected. Two independent reviewers will then use the CADIMA (Client-Server Application for Documentation and Integrated Management of All Steps of a Systematic Review) web tool to screen records for titles, abstracts, and full texts. Data will be extracted from eligible studies by 2 independent reviewers by using a data extraction sheet, including relevant information from the eligible studies helping to answer the research question. The data extraction sheet will be completed together with a quality assessment by using JBI and other relevant tools before the synthesis of all the relevant results. Numerical and visual results will be presented using descriptive summaries to organize, describe, and interpret the findings. We will also provide a narrative synthesis of the main results focused on our objectives. This scoping review is registered with the Open Science Framework.

**Results:**

The scoping review started in November 2024. The analysis is expected to be completed and the results submitted for publication in November 2025. The final review will report on all the studies identified and synthesized in accordance with the predefined protocol. Concerning all types of exposure, we expect to find more aspects of the specific external exposome (eg, pollutants, diet, infectious agents), particularly in young populations (children, adolescents) and possibly in utero exposition, linked to the trigger of the disease rather than in developing diabetes complications.

**Conclusions:**

By covering the different aspects and stages of the disease, this literature review aims to provide a comprehensive overview of the different effects of the components from the exposome in relation to T1D. This scoping review will help the scientific community understand the various aspects of the exposome in the development of T1D and its consequences. The results will be released as a peer-reviewed open-access publication and during congresses and conferences to reach the scientific community and the general public.

**Trial Registration:**

OSF Registries 10.17605/OSF.IO/UJ5KM; https://osf.io/uj5km

**International Registered Report Identifier (IRRID):**

DERR1-10.2196/73424

## Introduction

### Rationale

Type 1 diabetes (T1D) is a chronic autoimmune disease characterized by the destruction of the insulin-producing pancreatic β-cells, leading to absolute insulin deficiency and manifested by the sudden onset of severe hyperglycemia leading to lifelong dependence on exogenous insulin [1]. Over the past few decades, the incidence of T1D has risen significantly worldwide and cannot be explained by genetic changes alone; it is most likely attributable to environmental influences as well [2,3]. Although lifestyle and behavioral factors are known to contribute to health and disease [4-6], growing evidence suggests that other environmental exposures may also play a critical role in the risk of chronic diseases and associated complications such as diabetes [7,8].

Emerging research highlights the complex role of the exposome, defined as the totality of environmental exposures experienced by an individual from conception onwards [9]. This exposome includes various dimensions that interact over time to influence individuals’ health. It comprises the general external exposome (eg, built environment, social environment, physicochemical exposures), the specific external exposome (eg, diet, pollutants, financial status), and the internal exposome (eg, metabolic processes, microbiome). These domains are interconnected, with overlapping exposures acting cumulatively to drive disease risk and progression throughout life [10-12].

Many studies have provided valuable insights into the exposome's significant role in developing and managing chronic diseases [13]. For instance, a review found that cardiometabolic risk factors and cardiovascular disease have been linked primarily to poor air quality [14] and a wide range of other elements of the exposome [15]. In another review, ischemic heart disease progression has been linked to several dimensions from the exposome, such as air, light, acoustic pollution, soil pollution, water contamination, climate change, social stress, and infectious diseases [16]. The effect of components from the exposome such as pollutants, industrial and agricultural chemicals, toxins, lifestyle and social factors, diet, microbiome, and infection have also been reported for Alzheimer disease as well as other major neurodegenerative diseases, including Parkinson disease, amyotrophic lateral sclerosis, and multiple sclerosis [17-19]. Similarly, some studies have identified links between air pollution and heavy metals and susceptibility to the development and progression of chronic kidney disease, while associations with other dimensions from the exposome, such as chemicals and the built environment, remain understudied [20]. In the diabetes field, research has predominantly focused on type 2 diabetes (T2D) and gestational diabetes mellitus. Studies have shown exposure to ambient air pollution, persistent organic pollutants, and endocrine-disrupting chemicals play a critical role in the development of gestational diabetes mellitus [21], as well as air pollution, residential noise, and socioeconomic deprivation in the alteration of β-cells, leading to an increased risk of T2D [11,22].

Despite these interesting findings, the role of the exposome in T1D remains largely unexplored. We performed an explanatory search in relevant databases such as MEDLINE, Cochrane Database of Systematic Reviews, Open Science Framework, and PROSPERO. We did not identify current or underway scoping reviews on the exposome and its relationship to the development of T1D and its outcomes such as glycemic variability, long-term complications, and quality of life. Previous reviews have focused on specific exposures such as prenatal pollutants, diet, or viral infections [23-26]. However, the overall knowledge of the exposome remains dispersed. Even less is known about how these environmental factors impact individuals already living with T1D. Such a comprehensive understanding is essential for the identification of new strategies for disease prevention and management.

Conducting a scoping review is well-suited for this broad research question, as it allows for an exhaustive exploration, particularly for a fragmented research area. Unlike systematic reviews, scoping reviews map existing knowledge, identify research gaps, and comprehensively synthesize the available evidence [27-29]. This approach is beneficial for investigating the various environmental exposures in T1D development and its association with T1D disease outcomes, which remain underexplored.

### Objectives

The purpose of this protocol study is to describe the methodology for a scoping review assessing the current understanding of the role of the exposome in T1D development and complications. In the scoping review study, we aim to describe the main components of the exposome linked to T1D, report the most effective ways to collect exposome data, and highlight the existing gaps linked to this research question.

## Methods

### Protocol and Registration

This protocol was previously registered using the Open Science Framework Registries platform [30]. The scoping review will follow the Joanna Briggs Institute (JBI) methodology for scoping reviews [29,31] and the PRISMA-ScR (Preferred Reporting Items for Systematic Reviews and Meta-Analyses for Scoping Reviews) guidelines, shown in Multimedia Appendix 1 [32]. In addition, we will follow the guidance of the free web-based tool CADIMA (Client-Server Application for Documentation and Integrated Management of All Steps of a Systematic Review), which will be used to perform this review. CADIMA is designed to assist in conducting systematic reviews and evidence syntheses, including scoping reviews. It provides a structured workflow to manage and document the review process, facilitate collaboration, and ensure transparency and reproducibility. For a scoping review, CADIMA allows the importation of a reference list from a preliminary literature search and deduplicates records, defines eligibility criteria, screens studies (based on titles and abstracts, and full texts), extracts relevant data, and generates visual reports such as PRISMA diagrams [33].

### Research Question

JBI proposes the PCC (Population/Concept/Context) framework to determine the key notions of the research question [29,31,34]. It has been determined based on the elements in Table 1 and defined as follows: which and how could environmental exposures lead to the development of the disease or complications of the disease for those who already have T1D?

**Table 1:** PCC (Population/Concept/Context) framework for the definition of the research question.

PCC element	Definition	Example
Population	People with T1D^a^ or people who developed or will develop T1D	N/A^b^
Concept	Impact of the exposome on T1D development and on the complications in people who already have T1D	General external exposome Socioeconomic status, living conditions, climate, weather, etc
		Specific external exposome Work environment, chemical and physical exposure, biological and infectious agents, lifestyle, etc
		Internal exposome Biological processes, metabolism, etc
Context	Exposures on a lifespan perspective	In utero, childhood, adolescence, adulthood, older adult life; community or hospital setting

^a^T1D: type 1 diabetes.

^b^N/A: not applicable.

### Information Sources and Search

Before using the CADIMA tool, an exhaustive search for studies in various databases will be performed to identify a comprehensive corpus of relevant studies: PubMed, Embase (Elsevier), CINAHL (EBSCO), Web of Science (Clarivate), and Google Scholar, based on the key concepts of T1D and exposome. Key concepts and further ones will be associated using the classic syntactic techniques provided by PubMed. The search strategy, including all identified keywords and index terms, will be adapted for each database after several attempts to provide the most efficient source of information. An example of what will be done in PubMed as well as the strings adapted to other databases is shown in Multimedia Appendix 2. There will be no selection by language. Studies available only in the original language other than English will be translated using artificial intelligence tools and verified with native speakers when needed. Each time a paper will have to be translated in English, a native-speaking scientist from our institution will be involved to check the relevance of the translation. Additional search strategies will be employed for additional sources by using backward and forward snowballing approaches, citations from review studies, and alerts for new studies from the different database queries.

### Selection of Sources of Evidence

Following the literature search, all identified studies concerning the impact of environmental exposures on the development or outcomes of T1D will be collected. All studies will be uploaded to the CADIMA tool, and duplicates will be removed. Two independent reviewers will perform the screening for titles and abstracts. After that, 2 independent researchers will select the studies by full text before extracting the data and summarizing all the relevant findings. The study eligibility criteria will be defined as follows.

1. Population: people with T1D or people who developed or will develop T1D.
2. Outcome: glycemic control variables; acute (ketoacidosis, hypoglycemia events) and chronic T1D complications (retinopathy, nephropathy, neuropathy, cardiovascular disease, and others); T1D development; T1D.
3. Exposome: general external exposome (eg, socioeconomic status, living conditions, climate, weather), specific external exposome (eg, work environment, chemical and physical exposure, biological and infectious agents, lifestyle) and internal exposome (eg, biological processes, metabolism), measured with sensors if applicable.
4. Exclusion criteria: no distinction between T1D and T2D, drug-induced glucose alterations (medications), review, case study.

As this work will focus on T1D, we will exclude studies on T2D or those where it is impossible to distinguish between T1D and T2D. For mixed populations, only results concerning T1D will be extracted. Studies that do not distinguish between T1D and T2D may introduce confounding factors, given their distinct etiology and pathophysiology [35-37]. The same applies to alterations in glucose levels caused by medication, which may lead to a bias in the interpretation of results, as they are not representative of the typical glycemic variability in people with T1D [38]. Finally, review studies will be excluded as they do not provide original data or specific methodologies. The same applies to case studies with limited scope for generalization [39].

Following a pilot test (consistency check to determine the agreement between reviewers when applying the selection criteria), 2 independent reviewers will screen titles and abstracts to assess them according to the study eligibility criteria defined for the review. CADIMA automatically and randomly allocates the studies to team members. The preliminary eligibility of the studies will be assessed at this stage. The selected studies by title and abstract will be evaluated for eligibility by full text. Authors of studies for which the full text is unavailable will be contacted using various communication channels (ResearchGate, email, LinkedIn) to maximize the chances of accessing the full text. During this stage, full texts will be excluded based on the eligibility criteria and for other reasons, such as being inaccessible or not presenting primary data/summary statistics. Any disagreements between the reviewers at each stage of the selection process will be solved through discussion among the reviewers, or if no consensus is reached, by involving a third reviewer. The search results and the study selection process will be fully documented in the final scoping review and presented as a PRISMA flow diagram.

### Data Items

Data will be extracted from eligible papers selected for the scoping review by 2 independent reviewers by using a data extraction sheet developed with the CADIMA tool. A draft of the data to be gathered is detailed in Multimedia Appendix 3. The data extracted will include comments, article ID, study ID, author, year of publication, title, data location, and study name as predefined by the CADIMA tool (columns 1 to 8, Multimedia Appendix 3) and additional columns defined by team members according to the research question (columns 9 to 62, Multimedia Appendix 3). This draft data extraction tool will be modified and revised as necessary during the data extraction process for each eligible study. Any disagreements between the reviewers will be solved through discussion.

### Critical Appraisal of Individual Sources of Evidence

The quality of the selected papers will be assessed by 2 independent reviewers by using JBI Critical Appraisal Tools [40] when provided for the specific study designs (eg, analytical cross-sectional, case-control, cohort studies, qualitative research, quasi-experimental, randomized controlled trials) and other relevant tools validated in the literature for study designs not covered by JBI, such as the National Institutes of Health and STROBE (Strengthening the Reporting of Observational studies in Epidemiology) checklists [41,42].

### Synthesis of Results

We will perform an analytical approach based on the following PCC (JBI) approach [34].

1. For the population, we will record the mean age, sex distribution, and country.
2. For the concept, we will identify outcomes and exposures by specifying the type of outcome (T1D development or T1D complication) and the type of exposome by grouping according to their nature, the assessment tool.
3. For the context, we will code according to the period of exposure studied.

To ensure that the findings respond correctly to the review question and objectives and to organize and interpret the data, we will use numerical summaries and thematic synthesis [43]. To provide an overview of the included studies, we will describe the data with numerical summaries. We will present the sample characteristics such as the year of publication, geographical location, study design, and population. The exposures will be detailed in terms of exposure type (eg, environmental pollutants, occupational exposures, lifestyle factors) and exposure period (eg, in utero, childhood, adulthood). This summary will help identify current trends and gaps in the existing literature. We will also classify using thematic synthesis. This classification will provide key dimensions of the exposome based on its consensual definition about the general external exposome, specific external and internal exposome, and its associations with T1D. The results will be presented according to the outcome definition (development of T1D in the general population vs complications and outcomes in people living with T1D). We will use descriptive tables to summarize the characteristics of the studies and figures or diagrams to illustrate the distribution of the exposome dimensions related to T1D and the development and complications of T1D.

## Results

Data collection for this scoping review began in November 2024. The completion of analysis and submission of results for publication are planned after 12 months from initiation. The findings will be reported as described above and submitted for publication in a peer-reviewed journal. Considering previous reviews, we anticipate finding papers that study single exposures rather than their interactions within the exposome. It is similarly possible that many more studies will focus on the effect of these exposures or of the exposome on the development of T1D. We also expect to have many more studies combining exposures when analyzing pollutants or air pollution, nutrition or else in utero/birth factors than combinations of other exposome types. Regarding the exposome analysis methodology, it could vary from very classical analysis to comprehensive and sophisticated methods, including machine learning and exposome-wide association studies.

## Discussion

### Summary of Evidence

This scoping review aims to discuss in detail the current knowledge of the impact of the exposome in the context of T1D by exploring its role in the development of the disease and its subsequent complications. We expect that the principal findings will mainly concern aspects from the specific external exposome (eg, diet, infectious agents, pollutants). We expect to find more studies on the development of T1D (in young populations and possibly in utero exposure) rather than research on T1D complications. Indeed, several reviews and meta-analyses have already highlighted specific components of the exposome and their role in the development and the evolution of the disease [44-47]. Moreover, the current research seems to focus on a single impact of specific exposures rather than the combined effect, acting as a trigger at an early stage of the disease, particularly during onset. Although these studies have led to a better understanding of how the environment can affect diabetes, further aspects of the exposome and its relationship with T1D remain underexplored. To our knowledge, this is the first literature review to explore the role of the exposome in both the development of T1D and its complications in individuals living with T1D.

### Strengths and Limitations

The principal strength of this review lies in its comprehensive scope. It will provide an in-depth examination of a recent and evolving concept while covering stages of the disease. However, this approach also implies that some limitations are expected. First, as the exposome concept is new and evolving, its dimensions are not always well-defined. Additionally, the measurement methods for environmental exposures could vary a lot from objective to subjective methods, and because of this heterogeneity, it could make it challenging to summarize and define the most effective ways to collect them. Lastly, it could be difficult to interpret the findings due to the different outcomes studied in this review, including the development of the disease or its complications.

### Conclusions

In conclusion, this review protocol aims to establish a methodology for a comprehensive examination of the impact of the exposome in T1D and to highlight the existing gaps in the current research. The findings are expected to provide a valuable resource for future research projects in the development of personalized strategies for the prevention and management of T1D. In order to give the broadest possible access to this research work, the results of this scoping review will be disseminated via several channels such as an open-access paper, presentations at congresses or conferences on the topic, or press releases aimed at the scientific community and the general population.

## Appendix

Multimedia Appendix 1 PRISMA-ScR checklist.

Multimedia Appendix 2 Search strategy.

Multimedia Appendix 3 Data extraction sheet.

## Abbreviations

CADIMA: Client-Server Application for Documentation and Integrated Management of All Steps of a Systematic Review
JBI: Joanna Briggs Institute
PCC: Population/Concept/Context
PRISMA-ScR: Preferred Reporting Items for Systematic Reviews and Meta-Analyses for Scoping Reviews
STROBE: Strengthening the Reporting of Observational studies in Epidemiology
T1D: type 1 diabetes
T2D: type 2 diabetes

## Data Availability

All the findings from this scoping review will be made available through peer-reviewed open-access publication. All relevant documentation relating to the review will be stored in the Open Science Framework project, which has been registered under the following DOI: 10.17605/OSF.IO/UJ5KM.
