# Supplementary material for: Impact of the Exposome in Type 1 Diabetes: Protocol for a Scoping Review
Source: JMIR Res Protoc. 2025 Jul 17;14:e73424. doi: 10.2196/73424 (PMC12314464; doi:10.2196/73424)
Supplement: Multimedia Appendix 2 [file resprot_v14i1e73424_app2.docx]

### Appendix 2: Search strategy

| *Key concepts* | *Search terms/Strings in PubMed* | *Further concepts* | *Search terms/Strings in PubMed* |
| --- | --- | --- | --- |
| *T1D* | *(Diabetes Mellitus, Type 1[MeSH Terms] OR T1D OR "type 1 diabetes")* | *N/A* | *N/A* |
| *Exposome* | *Exposome[Mesh] OR exposome OR "Environmental Exposure"[Mesh] OR (Environment AND expose)* | *Biological and infectious agents* | *((Infection[Mesh] OR Antigen[Mesh] OR Allergen[Mesh] OR Pathogen* OR pathogens OR allergens OR "infectious agents" OR bacteria OR viruses OR "parasitic infections"))* |
|  |  | *Chemical and physical exposure* | *(("Environmental Pollution"[Mesh] OR "Air Pollutants"[Mesh] OR "water contaminants" OR pesticides OR "chemical exposures" OR Pollution OR pollutant* OR pesticide* OR Chemical OR "air pollution" OR "water contamination" OR "endocrine disruptors" OR "Dietary exposure"[Mesh] OR "food additives" OR nois* OR "light pollution"[Mesh] OR "Radiation"[Mesh] OR "physical exposures" OR agriculture OR "land cover" OR "land use" OR "heavy metals" OR "toxic dump" OR "storage sites"))* |
|  |  | *Climate and Weather* | *(("Climate"[Mesh] OR climat* OR climate changes OR "Meteorological Concepts"[Mesh] OR "weather patterns" OR weather OR "meteorological factors" OR temperature OR humidity OR "air pressure"))* |
|  |  | *Lifestyle* | *((Lifestyle[Mesh] OR smoking OR "alcohol consumption" OR "drug use" OR "physical activity" OR diet OR sleep))* |
|  |  | *Living conditions* | *(("Urbanization"[Mesh] OR "urban environment" OR "urban areas" OR "urban sprawl" OR "population density" OR "Neighborhood Characteristics"[Mesh] OR rural OR "green space*" OR "greenspace*" OR "housing quality" OR "living conditions" OR "built environment"))* |
|  |  | *Socioeconomic status* | *(("Socioeconomic Factors"[Mesh] OR "socioeconomic status" OR "socio-economic status" OR "socioeconomic factors" OR "income level" OR "educational attainment" OR "educational level" OR "employment status"))* |
|  |  | *Work environment* | *(("Occupational Exposure"[Mesh] OR "work environment" OR "occupational stress" OR workplace OR "working hours" OR "Working Conditions"[Mesh] OR "employment"[Mesh] OR "work commute"))* |
|  |  | *Data measurements (sensors)* | *(("Sensor Technology" OR "Wearable Sensors" OR "Environmental Sensors" OR "health app" OR "Continuous Glucose Monitor" OR cgm OR accelerometer OR "Global Positioning System" OR GPS OR "Geographic Information Systems" OR GIS OR smartwatch))* |
| *Databases* | *Adapted Search terms/Strings* | | |
| *Embase (Elsevier)* | *('insulin dependent diabetes mellitus'/exp OR 't1d' OR 'type 1 diabetes') AND (('exposome'/exp OR 'exposome' OR 'environmental exposure'/exp OR ('environment' AND 'expose')) AND ((('socioeconomics'/exp OR 'socioeconomic status' OR 'socio-economic status' OR 'socioeconomic factors' OR 'income level' OR 'educational attainment' OR 'educational level' OR 'employment status')) OR (('urbanization'/exp OR 'urban environment' OR 'urban areas' OR 'urban sprawl' OR 'population density' OR 'neighborhood characteristic'/exp OR 'rural' OR 'green space*' OR 'greenspace*' OR 'housing quality' OR 'living conditions' OR 'built environment')) OR (('climate'/exp OR 'climat*' OR 'climate changes' OR 'meteorological phenomena'/exp OR 'weather patterns' OR 'weather' OR 'meteorological factors' OR 'temperature' OR 'humidity' OR 'air pressure')) OR (('occupational exposure'/exp OR 'work environment' OR 'occupational stress' OR 'workplace' OR 'working hours' OR 'work environment'/exp OR 'employment'/exp OR 'work commute')) OR (('pollution'/exp OR 'air pollutant'/exp OR 'water contaminants' OR 'pesticides' OR 'chemical exposures' OR 'pollution' OR 'pollutant*' OR 'pesticide*' OR 'chemical' OR 'air pollution' OR 'water contamination' OR 'endocrine disruptors' OR 'dietary exposure'/exp OR 'food additives' OR 'nois*' OR 'light pollution'/exp OR 'radiation'/exp OR 'physical exposures' OR 'agriculture' OR 'land cover' OR 'land use' OR 'heavy metals' OR 'toxic dump' OR 'storage sites')) OR (('infection'/exp OR 'antigen'/exp OR 'allergen'/exp OR 'pathogen*' OR 'pathogens' OR 'allergens' OR 'infectious agents' OR 'bacteria' OR 'viruses' OR 'parasitic infections')) OR (('lifestyle'/exp OR 'smoking' OR 'alcohol consumption' OR 'drug use' OR 'physical activity' OR 'diet' OR 'sleep')) OR (('sensor technology' OR 'wearable sensors' OR 'environmental sensors' OR 'health app' OR 'continuous glucose monitor' OR 'cgm' OR 'accelerometer' OR 'global positioning system' OR 'gps' OR 'geographic information systems' OR 'gis' OR 'smartwatch'))))* | | |
| *CINAHL (EBSCO)* | *((MH "Diabetes Mellitus, Type 1" OR "type 1 diabetes" OR T1D) AND (MH "Exposome" OR exposome OR "environmental exposure" OR (environment AND expose))) AND ((MH "Socioeconomic Factors" OR "socioeconomic status" OR "income level" OR "educational attainment" OR "employment status") OR (MH "Urbanization" OR "urban areas" OR "green space" OR "population density" OR "housing quality" OR "living conditions") OR (MH "Climate" OR climat* OR "weather patterns" OR temperature OR humidity OR "air pressure") OR (MH "Occupational Exposure" OR "workplace" OR "working conditions" OR "work commute") OR (MH "Environmental Pollution" OR "air pollution" OR pollutants OR pesticides OR "water contamination") OR (MH "Infection" OR "pathogens" OR allergens) OR (MH "Lifestyle" OR smoking OR "alcohol consumption" OR diet OR sleep) OR ("sensor technology" OR "wearable sensors" OR "health app" OR "continuous glucose monitor" OR cgm OR "accelerometer" OR "GPS" OR "GIS" OR smartwatch) )* | | |
| *Web of Science (Clarivate)* | (ALL=(type 1 diabetes) OR ALL=(T1D) OR TS=(Diabetes Mellitus, Type 1)) AND (ALL=(exposome) OR ALL=("environmental exposure") OR ALL=("environment and expose")) AND ((ALL="socioeconomic status" OR ALL="socio-economic status" OR ALL="socioeconomic factors" OR ALL="income level" OR ALL="educational attainment" OR ALL="employment status") OR (ALL="urbanization" OR ALL="urban environment" OR ALL="urban areas" OR ALL="urban sprawl" OR ALL="population density" OR ALL="neighborhood characteristics" OR ALL=rural OR ALL="green space" OR ALL="greenspace" OR ALL="housing quality" OR ALL="living conditions" OR ALL="built environment") OR (ALL="climate" OR ALL=climat OR ALL="climate change" OR ALL="weather patterns" OR ALL=temperature OR ALL=humidity OR ALL="air pressure") OR (ALL="work environment" OR ALL="occupational stress" OR ALL=workplace OR ALL="working hours" OR ALL="work commute") OR (ALL="pollution" OR ALL="air pollutants" OR ALL="water contaminants" OR ALL=pesticides OR ALL=chemicals OR ALL="air pollution" OR ALL="water contamination" OR ALL="endocrine disruptors" OR ALL="food additives" OR ALL=noise OR ALL="light pollution" OR ALL=radiation OR ALL=agriculture OR ALL="land use" OR ALL="heavy metals" OR ALL="toxic dump") OR (ALL="infection" OR ALL=pathogens OR ALL=allergens OR ALL=bacteria OR ALL=viruses OR ALL="parasitic infections") OR (ALL="lifestyle" OR ALL=smoking OR ALL="alcohol consumption" OR ALL="drug use" OR ALL="physical activity" OR ALL=diet OR ALL=sleep) OR (ALL="sensor technology" OR ALL="wearable sensors" OR ALL="environmental sensors" OR ALL="health app" OR ALL="continuous glucose monitor" OR ALL=CGM OR ALL=accelerometer OR ALL=GPS OR ALL=GIS OR ALL=smartwatch)) NOT ALL=("type 2 diabetes") NOT ALL=(T2D) NOT TS=(Diabetes Mellitus, Type 2) NOT ALL=("Type II diabetes") | | |
| *Google Scholar* | T1D AND exposome environment "type 1 diabetes" | | |

### 
