# Supplementary material for: Impact of the Exposome in Type 1 Diabetes: Protocol for a Scoping Review
Source: JMIR Res Protoc. 2025 Jul 17;14:e73424. doi: 10.2196/73424 (PMC12314464; doi:10.2196/73424)
Supplement: Multimedia Appendix 3 [file resprot_v14i1e73424_app3.docx]

### Appendix 3: Data extraction sheet

| *Columns number* | *Category* | *Data type* |
| --- | --- | --- |
| *1-8* | *CADIMA* | *comments*  *article id*  *study id*  *author*  *publication year*  *title*  *data location*  *study_name* |
| *9-62* | *Study details* | *main_objective*  *secondary_objectives*  *study_type*  *study_design*  *country_of_study* |
|  | *Concept* | *islet_autoimmunity*  *T1D_development*  *T1D_complications_acute*  *T1D_complications_chronic*  *T1D_complications_other*  *other_description* |
|  | *Population* | *context (setting)*  *population_type*  *population_size_T1D*  *population_size_baby*  *population_size_children*  *population_size_adolescents*  *population_size_adults*  *population_size_elderly*  *control_size*  *min_max_age*  *mean_age*  *median_age*  *age_at_diagnosis*  *proportion_women*  *disease_susceptibility*  *family_history_DM*  *duration_T1D_mean*  *duration_T1D_median*  *exclusion criteria* |
|  | *Exposure* | *data_collection*  *exposition_period*  *exposure_SES*  *exposure_housing*  *exposure_mapping*  *exposure_work*  *exposure_climate*  *exposure_physical*  *exposure_chemicals*  *exposure_air_pollution*  *exposure_biological*  *exposure_lifestyle*  *exposure_pregnancy*  *parents_information* |
|  | *Outcomes* | *main_outcome*  *secondary_outcomes*  *outcomes_measures* |
|  | *Results* | *statistical_analysis*  *potential_confounders*  *tools_packages*  *findings*  *future_research* |
|  | *Other* | *limitations*  *linked_articles* |
